# 基于中国胸腺瘤协作组回顾性数据库对比Masaoka-Koga分期和国际肺癌协会/国际胸腺肿瘤协作组提出的TNM分期系统

**DOI:** 10.3779/j.issn.1009-3419.2016.07.04

**Published:** 2016-07-20

**Authors:** 光辉 梁, 志涛 谷, 印 李, 剑华 傅, 毅 沈, Yucheng WEI, 黎杰 谭, 鹏 张, 泳涛 韩, 椿 陈, 仁泉 张, 克能 陈, 和忠 陈, 永煜 刘, 有斌 崔, 允 王, 烈文 庞, 振涛 于, 鑫明 周, 阳春 柳, 媛 刘, 文涛 方, 中国胸腺肿瘤协作组成员

**Affiliations:** 1 200032 郑州，郑州大学附属肿瘤医院胸外科 Department of Thoracic Surgery, Affiliated Cancer Hospital of Zhengzhou University, Zhengzhou 450008, China; 2 200030 上海，上海交通大学附属上海胸科医院 Department of Thoracic Surgery, Shanghai Chest Hospital, Shanghai Jiao Tong University, Shanghai 200030, China; 3 510060 广州，中山大学附属肿瘤医院胸外科 Department of Thoracic Surgery, Guangdong Esophageal Cancer Institute, Sun Yat-sen University Cancer Center, State Key Laboratory of Oncology in South China, Collaborative Innovation Center of Cancer Medicine, Guangzhou 510060, China; 4 266001 青岛，青岛大学医学院附属医院胸外科 Department of Thoracic Surgery, Affiliated Hospital of Qingdao University, Qingdao 266001, China; 5 200032 上海，复旦大学附属中山医院胸外科 Department of Thoracic Surgery, Zhongshan Hospital, Fudan University, Shanghai 200032, China; 6 300052 天津，天津医科大学附属总医院胸外科 Department of Endocrinology, Tianjin Medical University General Hospital, Tianjin 300052, China; 7 610041 成都，四川省肿瘤医院胸外科 Department of Thoracic Surgery, Sichuan Cancer Hospital, Chengdu 610041, China; 8 350001 福州，福建医科大学附属协和医院胸外科 Department of Thoracic Surgery, Fujian Medical University Union Hospital, Fuzhou 350001, China; 9 230022 合肥，安徽医科大学附属第一医院胸外科 Department of Thoracic Surgery, First Affiliated Hospital of Anhui Medical University, Hefei 230022, China; 10 100142 北京，北京大学附肿瘤医院胸外科 Department of Thoracic Surgery, Beijing Cancer Hospital, Beijing 100142, Chin; 11 200433 上海，长海医院胸心外科 Department of Cardiothoracic Surgery, Changhai Hospital, Shanghai 200433, China; 12 110042 沈阳，辽宁肿瘤医院胸外科 Department of Thoracic Surgery, Liaoning Cancer Hospital, Shenyang 110042, China; 13 130021 长春，吉林大学附属第一医院胸外科 Department of Thoracic Surgery, First Affiliated Hospital of Jilin University, Changchun 130021, China; 14 610041 成都，四川大学华西医院胸外科 Department of Thoracic Surgery, West China Hospital, Sichuan University, Chengdu 610041, China; 15 200032 上海，复旦大学附属华山医院胸外科 Department of Thoracic Surgery, Huashan Hospital, Fudan University, Shanghai 200032, China; 16 300060 天津，天津医科大学附属肿瘤医院食管癌中心 Department of Esophageal Cancer, Tianjin Cancer Hospital, Tianjin 300060, China; 17 310022 杭州，浙江省肿瘤医院胸外科 Department of Thoracic Surgery, Zhejiang Cancer Hospital, Hangzhou 310022, China; 18 330006 南昌，江西省人民医院胸外科 Department of Thoracic Surgery, Jiangxi People's Hospital, Nanchang 330006, China

**Keywords:** 胸腺瘤, 分期, 预后分组, Thymoma, Staging, Prognostic grouping

## Abstract

**背景与目的:**

使用中国胸腺协作组（Chinese Alliance for Research in Thymomas, ChART）回顾性数据，比较Masaoka-Koga分期系统和国际肺癌协会（International Association for the Study of Lung Cancer, IASLC）/国际胸腺肿瘤协作组（International Thymic Malignancies Interest Group, ITMIG）推荐的新TNM分期对胸腺肿瘤预后的预测作用。

**方法:**

我们回顾分析了1992年-2012年ChART数据库共2, 370例患者。其中1, 198例信息完整的患者被纳入研究，按照TNM及Masaoka-Koga分期系统进行分期，并进行生存分析。评估指标为R0患者的累积复发率（cumulative incidence of recurrence, CIR）以及患者总生存率（overall survival, OS）。对比分析Masaoka-Koga分期系统和新的TNM分期系统。

**结果:**

根据Masaoka-Koga分期系统，不同分期CIR差异具有统计学意义，其中Ⅰ期和Ⅱ期或者Ⅱ期和Ⅲ期之间累积复发率无差异，Ⅳ期的患者具有更高的复发率，预后最差。根据新的TNM分期系统，T1a患者的累积复发率低于其他患者（*P* < 0.05），T1a患者总生存高于T1b患者（*P*=0.004），T4患者总生存率差于其它患者。N（+）患者累及复发率及总生存率差于N0患者。在M0患者与M1b患者之间总体累积复发率和总体生存率差异具有统计学意义，但在M0和M1b患者之间二者无差异。Ⅰ期-Ⅲa期与Ⅲb期-Ⅳb期患者之间总生存率具有差异，然而Ⅲb期与Ⅳb期患者之间总生存率无差异。

**结论:**

与Masaoka-Koga分期相比，IASLC/ITMIG TNM分期系统不仅描述了肿瘤侵犯的范围，也提供了有关淋巴结转移和肿瘤播散的情况。使用新的TNM分期系统进行前瞻性研究有助于更好的对胸腺肿瘤分组，预测预后，并指导治疗。

## Abstract (en)

**Background and objective:**

To compare the predictive effect of the Masaoka-Koga staging system and the International Association for the Study of Lung Cancer (IASLC)/the International Thymic Malignancies Interest Group(ITMIG) proposal for the new TNM staging on prognosis of thymic malignancies using the Chinese Alliance for Research in Thymomas (ChART) retrospective database.

**Methods:**

From 1992 to 2012, 2, 370 patients in ChART database were retrospectively reviewed. Of these, 1, 198 patients with complete information on TNM stage, Masaoka-Koga stage, and survival were used for analysis. Cumulative incidence of recurrence (CIR) was assessed in R0 patients. Overall survival (OS) was evaluated both in an R0 resected cohort, as well as in all patients (any R status). CIR and OS were first analyzed according to the Masaoka-Koga staging system. Then, they were compared using the new TNM staging proposal.

**Results:**

Based on Masaoka- Koga staging system, significant difference was detected in CIR among all stages. However, No survival difference was revealed between stage Ⅰ and Ⅱ, or between stage Ⅱ and Ⅲ. Stage Ⅳ carried the highest risk of recurrence and worst survival. According to the new TNM staging proposal, CIR in T1a was significantly lower comparing to all other T categories (*P* < 0.05) and there is a significant difference in OS between T1a and T1b (*P*=0.004). T4 had the worst OS comparing to all other T categories. CIR and OS were significantly worse in N(+) than in N0 patients. Significant difference in CIR and OS was detected between M0 and M1b, but not between M0 and M1a. OS was almost always statistically different when comparison was made between stages Ⅰ-Ⅲa and stages Ⅲb-Ⅳb. However, no statistical difference could be detected among stages Ⅲb to Ⅳb.

**Conclusion:**

Compared with Masaoka-Koga staging, the IASLC/ITMIG TNM staging proposal not only describes the extent of tumor invasion but also provides information on lymphatic involvement and tumor dissemination. Further study using prospectively recorded information on the proposed TNM categories would be helpful to better grouping thymic tumors for predicting prognosis and guiding clinical management.

迄今为止，胸腺肿瘤尚无被广泛采纳应用的分期系统，也无被国际癌症联盟（Union for International Cancer Control, UICC）定义的官方分期系统。由Koga等改良的Masaoka分期系统是目前相对应用最广泛的分期系统^[1, 2]^。虽然该分期系统在许多研究中表明与胸腺肿瘤的预后具有相关性^[3]^，但它仅仅建立在一个3 0年前单中心小样本（*n*=91例）研究之上。与大多数其他恶性肿瘤分期系统相比，Masaoka-Koga系统是不全面的，它不能像TNM分期那样将淋巴结转移或血液转移与肿瘤直接侵犯对预后的影响加以区分。因此，胸腺肿瘤需要一个建立在大样本数据之上的TNM分期来指导将来的实践和研究^[4]^。在胸腺肿瘤协作组（International Thymic Malignancy Interest Group, ITMIG）和国际肺癌协会（International Association for the Study of Lung Cancer, IASLC）联合协作下，胸腺肿瘤预后与分期专业委员会最近推荐了一个新的TNM分期系统^[5]^。在此，我们使用中国胸腺瘤协作组（Chinese Alliance for Research in Thymomas, ChART）回顾性数据库的资料对这两种分期系统进行比较。

## 1 材料与方法

我们回顾分析了1992年-2012年间ChART回顾性数据库中共2, 370例患者，数据库中患者来源于中国18个三级医疗中心。排除1, 172例患者（627例患者缺少信息不能进行TNM分期、2例患者不能进行Masaoka-Koga分期、543例患者缺失生存时间），1, 198例患者纳入分析。评估指标为R0患者累积复发率（cumulative incidence of recurrence, CIR）和患者总生存率（overall survival, OS）。对比分析Masaoka-Koga分期系统和新的TNM分期系统。

统计分析采用SPSS 18.0软件进行。生存分析采用*Kaplan-Meier*法，组间差异比较采用*Log-rank*检验。多因素分析采用*Cox*风险比例模型。*P*值采用双侧检验，*P* < 0.05被定为差异具有统计学意义。

## 2 结果

根据Masaoka-Koga分期，病理分期为Ⅰ期者618例，Ⅱ期200例，Ⅲ期319例、Ⅳa期23例、Ⅳb期38例。R0切除患者复发率随着肿瘤分期的增高而增加（表 1），同时所有患者的总体生存率随着分期的降低而减低（表 2）。R0切除患者的累积复发率见图 1和表 3。其中，Ⅰ期与Ⅱ期或Ⅲ期之间累积复发率差异有统计学意义（*P*值分别为*P*= 0.005，*P* < 0. 001），Ⅱ期和Ⅲ期之间差异亦有统计学意义（*P*= 0.007）。不同切除状态患者不同分期之间的总生存率比较见图 2和表 3，其中，对于任何切除状态的患者，Ⅰ期和Ⅲ期之间总生存率差异有统计学意义（*P* < 0.001），Ⅳb期患者与其他患者之间总生存率差异有统计学意义（*P* < 0.05）。然而Ⅱ期分别与Ⅰ期、Ⅲ期相比总体生存率差异无统计学意义（*P*值分别为*P*=0.111, *P*=0.103）。

**1:** 基于Masaoka-Koga分期R0患者复发或死亡总数的比例 Total proportion of recurrences or deaths of R0 patients base on Masaoka-Koga staging system

Masaoka-Koga	Recurrences			Deaths	
	%	*N*		%	*N*
Ⅰ	3	17/600		1	8/616
Ⅱ	6	12/197		2	4/197
Ⅲ	13	31/242		4	9/251
Total	6	60/1, 039		2	21/1, 064
注：本表得到版权所有者©2011-2016 Journal of Thoracic Disease复制许可。					

**2:** 基于Masaoka-Koga分期Rany患者复发或死亡总数的比例 Total proportion of recurrences or deaths of R any patients base on Masaoka-Koga staging system

Masaoka-Koga	Recurrences			Deaths	
	%	*N*		%	*N*
Ⅰ	3	17/600		1	8/616
Ⅱ	7	14/200		3	5/200
Ⅲ	16	49/308		5	16/319
Ⅳa	35	8/23		4	1/23
Ⅳb	32	12/38		24	9/38
Total	9	100/1,171		3	39/1,198
注：本表得到版权所有者©2011-2016 Journal of Thoracic Disease复制许可。					

**1:**
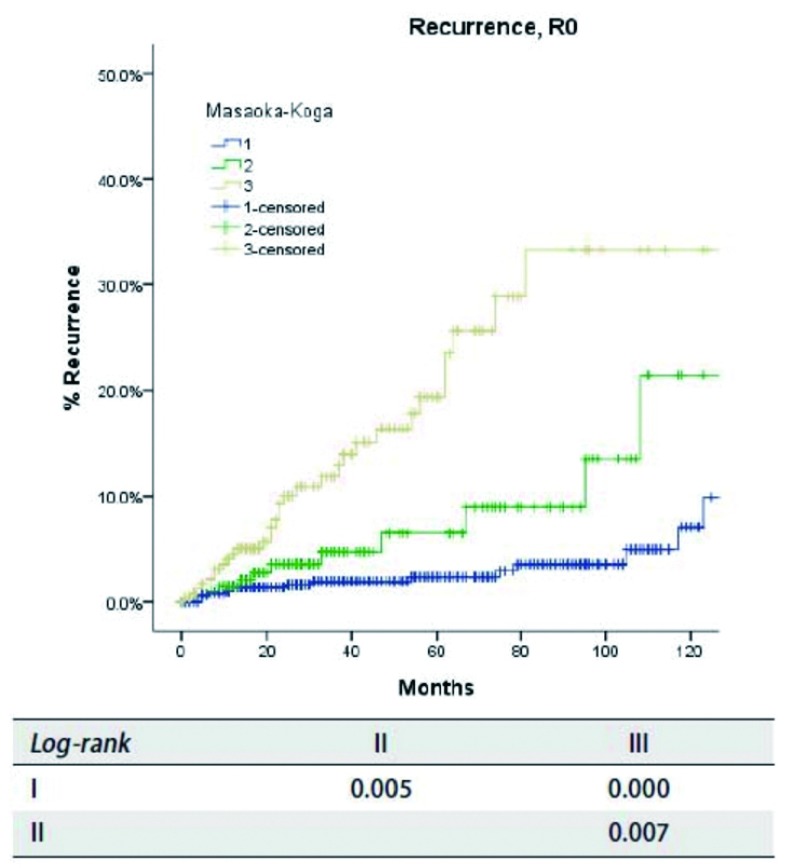
*Kaplan-Meier*生存曲线：按照Masaoka-Koga分期，R0切除不同分期患者的累积复发率(*Log-rank*)。 *Kaplan-Meier* survival curves: Cumulative recurrence rate of patients with R0 resection in different stage by the Masaoka-Koga staging (*Log-rank*).

**3:** Masaoka-Koga分期之间的差异 Differences between Masaoka-Koga categories

Variable	CIR, R0(67/1060)^a^			OS, R0(23/1085)^a^			OS, any R(39/1198)^a^	
	HR	*P*		HR	*P*		HR	*P*
HR *vs* adjacent Masaoka-Koga staging category								
Ⅱ *vs* Ⅰ	2.762	0.008		1.932	0.284		2.422	0.122
Ⅲ *vs* Ⅱ	2.428	0.009		1.904	0.286		2.265	0.113
Ⅳ *vs* Ⅲ	-	-		-	-		3.506	0.002
Ⅳb *vs* Ⅳa	-	-		-	-		6.482	0.078
Hazard ratios and statistical differences (*χ*^2^) by *Cox* proportional hazards regression models, adjusted by diagnosis. ^a^Number of events/total number of patients in entire data set for the particular analysis. CIR: cumulative incidence of recurrence; HR: hazard ratio; OS: overall survival; R0: complete resection. 注：本表得到版权所有者©2011-2016 Journal of Thoracic Disease复制许可。								

**2:**
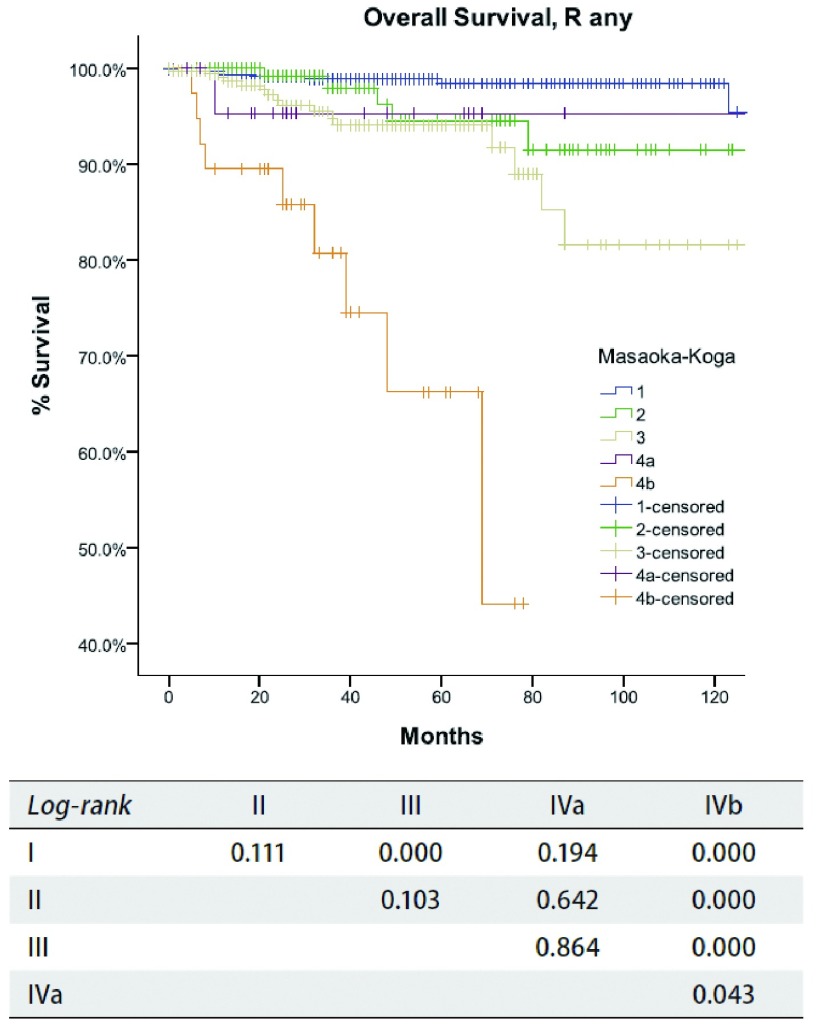
*Kaplan-Meier*生存曲线:按照Masaoka-Koga分期，任何R切除不同分期患者的总体生存期（*Log-rank*）。 *Kaplan-Meier* survival curves: Overall survival of patients with any R resection in different stage by the Masaoka-Koga staging (*Logrank*).

根据新的TNM分期方案，病理分期为Ⅰ期886例，Ⅱ期48例，Ⅲ期205例、Ⅳa期38例、Ⅳb期21例。R0切除患者复发率随着肿瘤分期的增高而增加（表 4），同时所有患者的总体生存率随着分期的降低而减低（表 5）。在分期为TxN0M0的R0切除患者中，T1a患者累积复发率低于其他T分期的患者（*P*＜0.05），值得注意的是T1a与T1b之间累积复发率差异亦有统计学意义（*P*=0.002, 1）。然而，T1b和T2或T3之间相比差异无统计学意义（*P*值分别为*P*=0.315，*P*=0.215），T2和T3之间的差异亦无统计学意义（*P*=0.963，图 3）。对于分期为TxN0M0的R0切除患者的总体生存率，T1a期患者高于T1b期患者（*P*= 0.004），而T1b期患者和T2或T3期相比无统计学差异（*P*值分别为*P*=0.428，*P*=0.481，图 4）。对于分期为TxN0M0的R1和R 2切除的患者，T4期患者总生存率低于其他T分期患者（*P*＜0.05，图 5）。*Cox*分析显示T1a期和T1b患者之间总生存率差异有统计学意义（*P* < 0.001），T3期和T4期患者之间总生产率差异亦具有统计学意义（*P*=0.001）；而T2期和T3期患者之间无统计学差异（*P*=0.72，表 6）。

**4:** 基于IASLC/ITMIG TNM分期的R0切除患者的复发或死亡总数的比例 Total proportion of recurrences or deaths of R0 patients base on the IASLC/ITMIG TNM staging proposal

Stage	Recurrences			Deaths	
	%	*N*		%	*N*
Ⅰ	4	32/858		2	14/874
T1aN0M0	4	28/792		1	11/808
T1bN0M0	6	4/66		5	3/66
Ⅱ	14	6/43		2	1/44
Ⅲa	16	22/134		4	6/142
Total	6	60/1, 035		2	21/1, 060
注：本表得到版权所有者©2011-2016 Journal of Thoracic Disease复制许可。					

**5:** 基于IASLC/ITMIG TNM分期的Rany切除患者的复发或死亡总数的比例 Total proportion of recurrences or deaths of R any patients base on the IASLC/ITMIG TNM staging proposal

Stage	Recurrences			Deaths	
	%	*N*		%	*N*
Ⅰ	4	36/870		2	17/886
T1aN0M0	4	30/798		1	12/814
T1bN0M0	8	6/72		7	5/72
Ⅱ	13	6/47		2	1/48
Ⅲ	19	38/195		5	11/205
Ⅲa	18	32/178		4	7/188
Ⅲb	35	6/17		24	4/17
Ⅳa	39	15/38		13	5/38
TxN1M0	43	6/14		29	4/14
TxN0M1a	36	8/22		5	1/22
TxN1M1a	50	1/2		0	0/2
Ⅳb	24	5/21		24	5/21
TxN2M0,1a	33	2/6		33	2/6
TxN0-2M1b	20	3/15		20	3/15
Total	9	100/1,171		3	39/1,198
注：本表得到版权所有者©2011-2016 Journal of Thoracic Disease复制许可。					

**6:** T分期的差异（IASLC/ITMIG TNM分期） Differences between T categories (IASLC/ITMIG TNM staging proposal)

Variable	CIR, R0 (60/1,039)^a^			OS, R0 (21/1,064)^a^			OS, any R (29/1,139)^a^	
	HR	*P*		HR	*P*		HR	*P*
HR *vs* adjacent T category								
T1b *vs* T1a	3.299	0.029		5.574	0.010		8.624	0.000
T2 *vs* T1b	1.898	0.323		0.41	0.443		0.266	0.227
T3 *vs* T1b	1.941	0.225		0.607	0.485		0.33	0.061
T2 *vs* T1	6.299	0		1.837	0.558		1.497	0.696
T3 *vs* T2	1.022	0.963		1.461	0.726		1.469	0.72
T4 *vs* T3	-	-		-	-		8.088	0.001
Hazard ratios and statistical differences (*χ*^2^) by *Cox* proportional hazards regression models, adjusted by diagnosis. ^a^Number of events/total number of patients in entire data set for the particular analysis. CIR, cumulative incidence of recurrence; HR, hazard ratio; OS, overall survival; R0, complete resection. 注：本表得到版权所有者©2011-2016 Journal of Thoracic Disease复制许可。								

**3:**
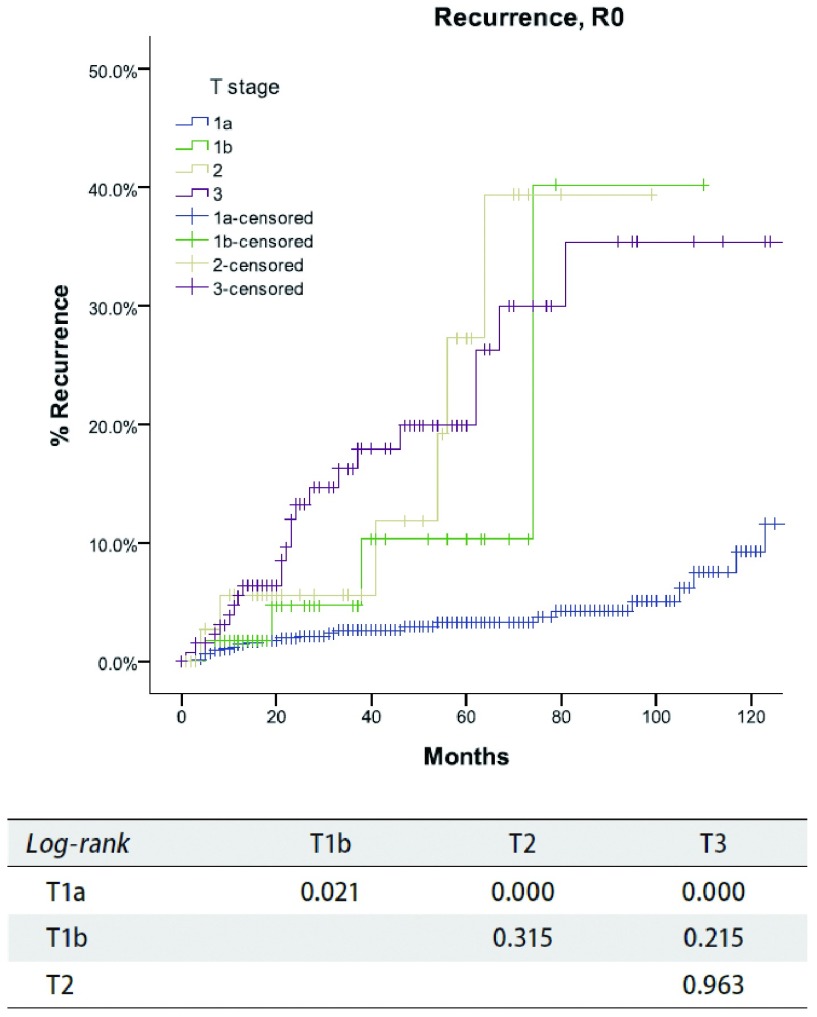
*Kaplan-Meier*生存曲线:按照IASLC/ITMIG TNM分期，R0切除的TxN0M0患者在不同T分期累积复发率（*Log-rank*）。 *Kaplan-Meier* survival curves: Cumulative recurrence rate of TxN0M0 patients with R0 resection in different T stage by the IASLC/ ITMIG TNM staging proposal (*Log-rank*).

**4:**
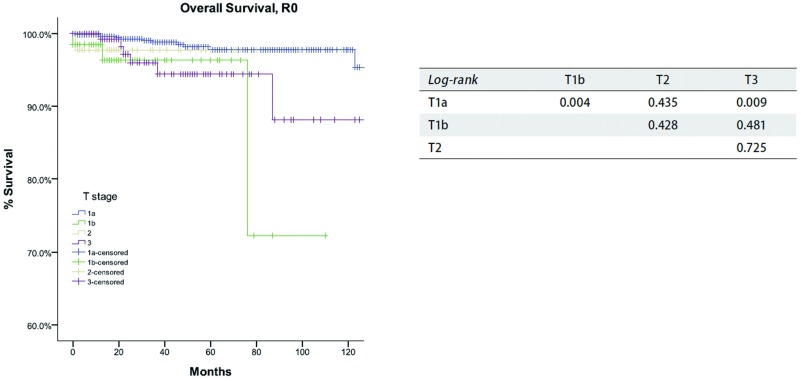
*Kaplan-Meier*生存曲线:按照IASLC/ITMIG TNM分期，R0切除的TxN0M0患者在不同T分期总体生存率（*Log-rank*）。 *Kaplan-Meier* survival curves: Overall survival of TxN0M0 patients with R0 resection in different T stage by the IASLC/ITMIG TNM staging proposal (*Log-rank*).

**5:**
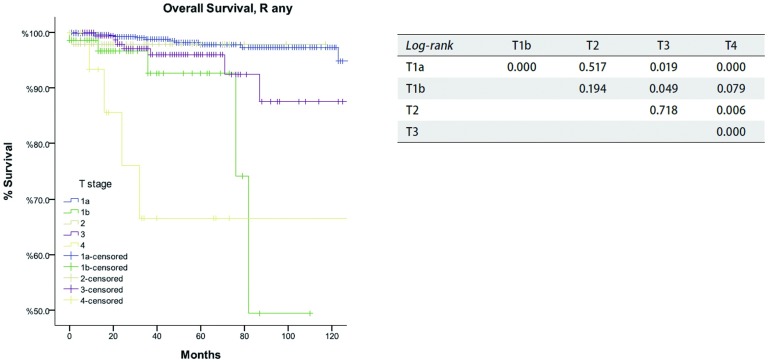
*Kaplan-Meier*生存曲线:按照IASLC/ITMIG TNM分期，Rany切除的TxN0M0患者在不同T分期总体生存率（*Log-rank*）。 *Kaplan-Meier* survival curves: Overall survival of TxN0M0 patients with R any resection in different T stage by the IASLC/ITMIG TNM staging proposal (*Log-rank*).

对于N分期，R0切除患者的累积复发率见图 6，所有患者的总体生存率见图 7。N0患者的累积复发率和总生存率均优于N（+）患者（*P* < 0.05），而N1和N2患者之间二者无统计学差异（*P* > 0.05）。*Cox*分析显示，N（+）患者累及复发率及总生存率均差于N0患者，是独立的不良预后因素（表 7）。

**6:**
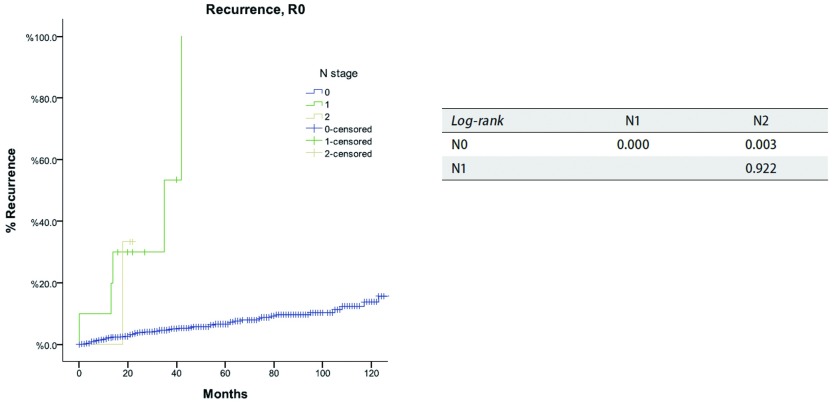
*Kaplan-Meier*生存曲线:按照IASLC/ITMIG TNM分期，R0切除的患者在不同N分期累积复发率（*Log-rank*）。 *Kaplan-Meier* survival curves: Cumulative recurrence rate of patients with R0 resection in different N stage by the IASLC/ITMIG TNM staging proposal (*Log-rank*).

**7:**
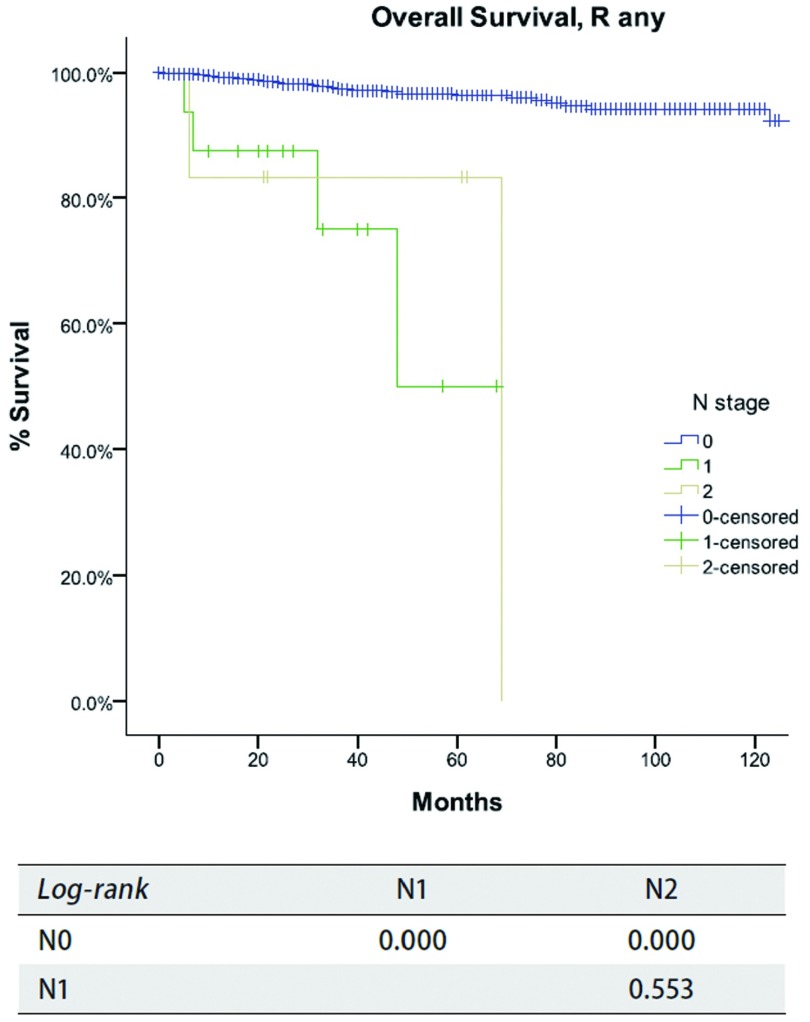
*Kaplan-Meier*生存曲线:按照IASLC/ITMIG TNM分期，R0切除的患者在不同N分期总体生存率（*Log-rank*）。 *Kaplan-Meier* survival curves: Overall survival of patients with R any resection in different N stage by the IASLC/ITMIG TNM staging proposal.

**7:** N分期的差异（IASLC/ITMIG TNM分期） Differences between N Categories (IASLC/ITMIG TNM staging proposal)

Variable	CIR, R0 (67/1,060)^a^			OS, R0 (23/1,085)^a^			OS, any R (39/1,198)^a^	
	HR	*P*		HR	*P*		HR	*P*
HR *vs* adjacent N category								
N1 *vs* N0	15.66	0.000		6.817	0.062		13.034	0.000
N2 *vs* N0	10.99	0.018		0.050	0.876		14.074	0.000
N2 *vs* N1	0.893	0.922		0.033	0.737		0.515	0.559
N1+N2 *vs* N0	14.77	0.000		4.968	0.119		8.617	0.000
Hazard ratios and statistical differences (*χ*^2^) by *Cox* proportional hazards regression models, adjusted by diagnosis. ^a^Number of events/total number of patients in entire data set for the particular analysis. 注：本表得到版权所有者©2011-2016 Journal of Thoracic Disease复制许可。								

对于M分期，M0患者的累积复发率低于M1患者（*P* < 0.05），而M1a和M1b患者之间累积生存率无统计学差异（*P*=0.263，图 8）。M0与M1a患者之间总生存差异无统计学意义（*P*=0.682，图 9），同样M1a和M1b之间亦无统计学差异（*P*=0.109）。

**8:**
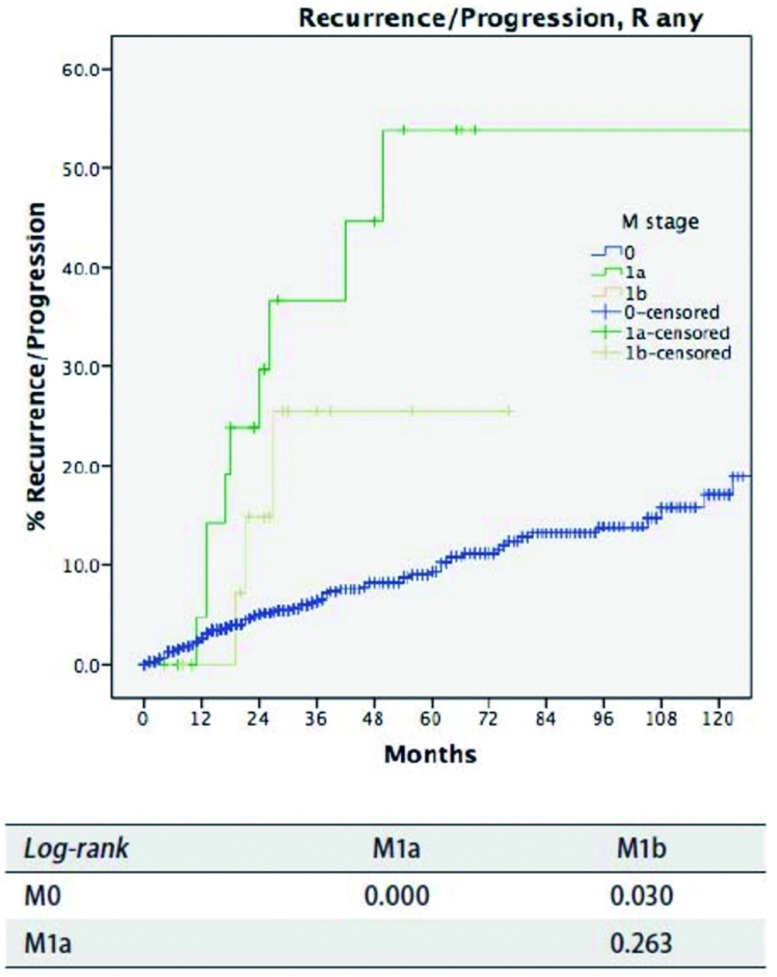
*Kaplan-Meier*生存曲线:按照IASLC/ITMIG TNM分期，Rany切除的患者在不同M分期累积复发率或进展率（*Log-rank*）。 *Kaplan-Meier* sur vival cur ves: Cumulative recurrence/ progression rate of patients with R any resection in different M stage by the IASLC/ITMIG TNM staging proposal (*Log-rank*).

**9:**
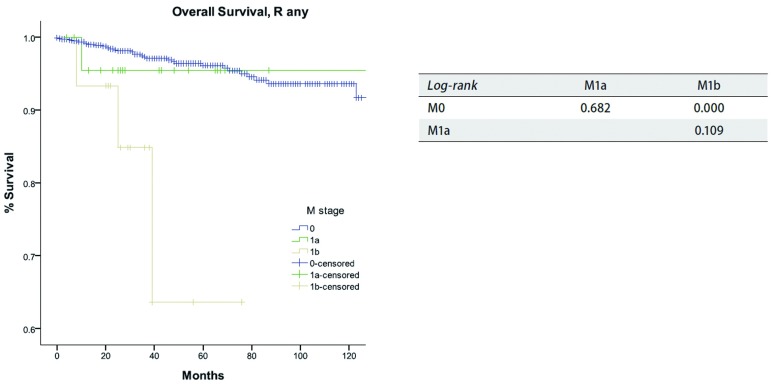
*Kaplan-Meier*生存曲线:按照IASLC/ITMIG TNM分期，Rany切除的患者在不同M分期总体生存率（*Log-rank*）。 *Kaplan-Meier* survival curves: Overall survival of patients with R any resection in different M stage by the 8th edition TNM staging (*Log-rank*).

基于所提出的新的TNM分期，Ⅰ期R0切除患者累积复发率低于Ⅱ期或Ⅲ期（*P*值分别为*P* < 0.001，*P* < 0.001），Ⅱ期和Ⅲa期之间差异无明显统计学意义（*P*=0.963）。所有患者中Ⅰ期和Ⅱ期患者之间总生存率无明显差异（*P*=0.694），Ⅱ期和Ⅲ期患者之间总生存亦无明显差异（*P*=0.718）。Ⅲa期患者的总生存率优于Ⅲb期患者（*P* < 0.001），Ⅳb期患者总生存率最差。此外，Ⅲb期和Ⅳa期患者之间总生存无统计学差异（*P* < 0.312），Ⅳa期和Ⅳb期之间也无统计学差异（*P* < 0.315）（图 10，表 8）。

**10:**
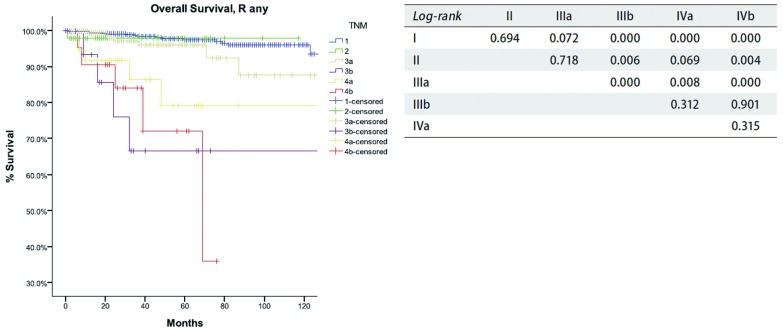
*Kaplan-Meier*生存曲线:按照IASLC/ITMIG TNM分期，Rany切除的患者在不同分期总体生存率（*Log-rank*）。 *Kaplan-Meier* survival curves: Overall survival of patients with any R resection in different stage by the IASLC/ITMIG TNM staging proposal (*Log-rank*).

**8:** IASLC/ITMIG TNM分期的差异 Differences between the IASLC/ITMIG TNM staging proposal categories

Variable	CIR, R0(67/1,060)^a^			OS, R0(23/1,085)^a^			OS, any R (39/1,198)^a^	
	HR	*P*		HR	*P*		HR	*P*
HR *vs* adjacent TNM staging category								
Ⅱ*vs*Ⅰ	0.159	0.000		0.544	0.558		1.497	0.696
Ⅲa *vs* Ⅰ	5.235	0.000		2.926	0.028		2.207	0.080
Ⅲb *vs* Ⅰ	-	-		-	-		16.665	0.000
Ⅳa *vs* Ⅰ	-	-		-	-		8.806	0.000
Ⅳb *vs* Ⅰ	-	-		-	-		17.847	0.000
Ⅲa *vs* Ⅱ	1.022	0.963		1.461	0.726		1.469	0.720
Ⅲb *vs* Ⅱ	-	-		-	-		11.282	0.030
Ⅳa *vs* Ⅱ	-	-		-	-		5.787	0.109
Ⅳb *vs* Ⅱ	-	-		-	-		12.108	0.024
Ⅲb *vs* Ⅲa	-	-		-	-		8.088	0.001
Ⅳa *vs* Ⅲa	-	-		-	-		4.209	0.015
Ⅳb *vs* Ⅲa	-	-		-	-		8.616	0.000
Ⅳa *vs* Ⅲb	-	-		-	-		0.515	0.323
Ⅳb *vs* Ⅲb	-	-		-	-		0.920	0.901
Ⅳb *vs* Ⅳa	-	-		-	-		1.872	0.322
Hazard ratios and statistical differences (*χ*^2^) by *Cox* proportional hazards regression models, adjusted by diagnosis. ^a^Number of events/total number of patients in entire data set for the particular analysis. 注：本表得到版权所有者©2011-2016 Journal of Thoracic Disease复制许可。								

## 3 讨论

胸腺肿瘤有大约10余种分期系统被提出^[6-17]^。但很少像其他大多数实体瘤那样采用了TNM分期方法。IASLC/ITMIG在Masaoka-Koga分期系统的基础上提出了胸腺肿瘤新的UICC分期-TNM分期。从表 9可以看出，在这个新的分期系统Ⅰ-Ⅲb期按照T分期划分，分别对应Masaoka-Koga系统Ⅰ-Ⅲ期。Ⅳa期由N1或M1a所决定，Ⅳb期由N2或M1b决定^[5]^，而在Masaoka-Koga分期系统中，所有淋巴结转移均划入Ⅳb期。

**9:** IASLC/ITMIG TNM分期和Masaoka-Koga分期的关系 The relationship between the IASLC/ITMIG TNM proposal staging categories and Masaoka-Koga staging system

The 8^th^ edition TNM stage	TNM	Definition (Involvement of)	Masaoka-Koga
Stage Ⅰ	T1aN0M0	Encapsulated or unencapsulated, with or without extension into mediastinal fat	Stage Ⅰ and Ⅱ
	T1bN0M0	Extension into mediastinal pleura	Stage Ⅲ (partial-pleura)
Stage Ⅱ	T2N0M0	Pericardium	Stage Ⅲ (partial-pericardium)
Stage Ⅲa	T3aN0M0	Lung, brachiocephalic vein, superior vena cava, chest wall, phrenic nerve, hilar (extrapericardial) pulmonary vessels	Stage Ⅲ (partial-completeness of resection)
Stage Ⅲb	T3bN0M0	Aorta, arch vessels, main pulmonary artery, myocardium, trachea, or esophagus	Stage Ⅲ (partial-incompleteness of resection)
Stage Ⅳa	TxN1M0	Anterior (perithymic) nodes	Stage Ⅳb
	TxN0M1afont	Separate pleural or pericardial nodule(s)	Stage Ⅳa
	TxN1M1a	Anterior (perithymic) nodes, Separate pleural or pericardial nodule(s)	Stage Ⅳb
Stage Ⅳb	TxN2M0	Deep intrathoracic or cervical nodes	Stage Ⅳb
	TxN2M1a	Deep intrathoracic or cervical nodes, Separate pleural or pericardial nodule(s)	Stage Ⅳb
	TxNxM1b	Pulmonary intraparenchymal nodule or distant organ metastasis	Stage Ⅳb
注：本表得到版权所有者©2011-2016 Journal of Thoracic Disease复制许可。			

我们的研究结果表明，虽然在Masaoka-Koga分期中，Ⅰ期至Ⅲ期患者间累积复发率存在差异，而Ⅰ期和Ⅱ期之间总体生存率是类似的（表 1-表 3，图 1-图 2）。这些表明Masaoka-Koga分期系统中Ⅰ期和Ⅱ期在ITMIG提出的分期系统中应归为T1a期（Ⅰ期）^[18]^。然而，有无侵犯到外膜或纵隔脂肪（Masaoka-Koga分期Ⅰ期和Ⅱ期）肿瘤复发率是有差异的，这需要我们探讨这部分分期是否需要进一步细分的问题，复发在惰性肿瘤中也是重要的指标^[19]^。

在Masaoka-Koga分期系统中，肿瘤侵犯纵隔胸膜被归为Ⅱ期和Ⅲ期。在IASLC/ITMIG提出的分期系统中归为Ⅰ期，因为纵隔胸膜侵犯与否在复发和总生存上均无统计学差异。日本胸腺协作组的已经发表的研究证实T1a和T1b之间的累积复发率具有差异，所以T1a和T1b被保留了下来。希望将来可以做进一步研究。在本组研究中，T1aN0M0和T1bN0M0患者之间的累积复发率和总生存率差异具有统计学意义（表 4，图 3-图 5）。理论上，胸膜侵犯增加胸膜腔扩散的机会，这也是胸腺肿瘤复发最常见的类型。鉴于病理上识别胸膜侵犯难度大，手术标本中标出纵隔胸膜是极其重要的，另外前瞻性记录浸润的情况对未来的研究也是极其重要的。

Masaoka-Koga分期系统中的Ⅲ期具有高度异质性。肿瘤侵犯纵隔胸膜（T1b），心包（T2），或任何其他结构（T3-4）都包含在一个单独类别中。在本组研究中，Masaoka-Koga分期中的Ⅱ期和Ⅲ期之间总生存无明显差异（表 1-表 3和图 1-图 2）。直观地说有限的外侵发生在容易切除的结构和不容易切除的重要器官对预后的影响不同。本组研究中，T1b至T3之间（IASLC/ITMIG提出的分期Ⅰ期至Ⅲa期）的累积复发率和总体生存率无差异（表 4-表 6和图 3-图 5）。T1与T2和T3之间复发或生存曲线是分离的可能是由于T1a患者预后更好造成。只有T4的患者（Ⅲb）生存和复发的结果最差。Ⅰ期与Ⅱ期或Ⅲa期患者之间总生存和复发无明显差异（表 8和图 10）。这与许多以前的研究相一致，揭示了完整的胸腺肿瘤切除是胸腺肿瘤的独立预后因素^[20]^。胸腺肿瘤全身性扩散是很少见的，只要病变可以完全切除，均能有良好预后。考虑到TNM系统是一个解剖分类，根据IASLC/ITMIG分期的T分期区分肿瘤侵犯程度是必要的。然而，预后分组仍应根据患者的长期随访结果。因此除了Ⅲb期（T4），对IASLC/ITMIG新分期进一步验证是有必要的。

所有胸腺肿瘤分期中，仅四项研究使用了TNM分期法^[11, 12, 15, 21]^。在所有分期中，淋巴结受累都被简单地认为是晚期肿瘤的标志。在IASLC/ITMIG提出的分期中，淋巴结转移也被划分到Ⅳ期。同时ITMIG提出了一个新的纵隔淋巴结图^[21]^。在新的分期中该图有助于划分N0至N1-2^[22]^。本组研究中总体生存率及累积复发率在N1和N2之间无明显差异，目前的研究在两种淋巴结状态之间也没有发现任何有意义的统计学差异，这可能是因为淋巴结阳性的患者数量相对较少，从而影响了生存分析的结果（表 7）。然而，我们的确发现淋巴结阳性患者累积复发率高于淋巴结阴性患者（图 6）及总体生存率明显降低（图 7）。淋巴结清扫术是治疗胸腺肿瘤的一个必要部分。如果缺少系统性淋巴结清扫或取样，正确评估淋巴结受累状态是不可能的。未来只有按以上规范的淋巴结处理收集方法为基础的研究，才可以正确评估淋巴结受累对预后的影响。

在IASLC/ITMIG推荐的分期中，M分为M1a（胸腔播散）和M1（远处转移）^[22]^。它们分别归为Ⅳa期和Ⅳb期，类似于Masaoka-Koga分期系统中Ⅳa和Ⅳb期。虽然M1a和M1b患者的生存曲线是分开的，但二者之间的累积复发率或总体生存率差异无统计学意义。当然二者预后好于M0患者（图 8和图 9）。另外，虽然M1a患者的累积复发率高于M0患者（图 8），但在总体生存率上无明显差异（图 9）。这可能再次归因于生存分析中小概率事件。胸腺肿瘤是一种惰性生长的肿瘤，即使当前肿瘤局部出现扩散如胸膜转移，仍有望长期生存。但远处转移确为不良预后因素之一。无论累积复发率和总体生存率，M1b组患者均差于M0患者。

根据预后分组，Ⅰ期-Ⅲa期与Ⅲa期-Ⅳb期患者之间总生存具有统计学差异（表 8和图 10）。Ⅰ期和Ⅲa期，Ⅱ期和Ⅳa期患者之间生存有差异，但未达到统计学意义（*P*值分别为0.072，0.069）。然而，Ⅲb期与Ⅳb期未发现明显统计学差异。尽管Ⅰ期累积复发率低于Ⅱ期或Ⅲa期患者，但三者之间总生存无统计学差异。

## 4 结论

综上所述，IASLC/ITMIG对胸腺肿瘤提出的新分期是对这种少见肿瘤认识前进的重要的一步。这是第一次基于一个全球合作的多中心数据进行了系统分析，采用TNM分期描述肿瘤的侵袭以及播散。在晚期疾病中，不能区分生存差异，主要是因为数据库的性质是以手术为主的，以及疾病本身具有进展缓慢和生存期长等特点。我们使用的ChART数据库也是以手术为主的，除了N0和N（+）或M0和M1之间有明显差异之外，未发现N1和N2或M1a和M1b之间的预后差异。在T分期中，T1a和T4代表了预后的两个极端，而T1b期至T3期之间对比显示复发或总体生存率无统计学差异。这反映了完全切除在胸腺肿瘤中的重要作用。新分期方案为未来更好的预后分组的研究提供了一个有用的工具。前瞻性的在每个病例中详细记录TNM分期的不同部分（T分期，N分期，M分期），有助于揭示它们的预后意义，这是无法通过回顾性研究获得的。
