# 更正声明

**DOI:** 10.3779/j.issn.1009-3419.2016.09.14

**Published:** 2016-09-20

本刊2016年第19卷第7期刊登的题为“基于中国胸腺瘤协作组回顾性数据库对比Masaoka-Koga分期和国际肺癌协会/国际胸腺肿瘤协作组提出的TNM分期系统”[梁光辉，谷志涛，李印，傅剑华，沈毅，谭黎杰，张鹏，韩泳涛，陈椿，张仁泉，陈克能，陈和忠，刘永煜，

崔有斌，王允，庞烈文，于振涛，周鑫明，柳阳春，刘媛，方文涛，中国胸腺肿瘤协作组成员.基于中国胸腺瘤协作组回顾性数据库对比Masaoka-Koga分期和国际肺癌协会/国际胸腺肿瘤协作组提出的TNM分期系统.中国肺癌杂志, 2016, 19(7): 425-436.]一文中:

1.第425页，中文作者应为“梁光辉，谷志涛，李印，傅剑华，沈毅，魏煜程，谭黎杰，张鹏，韩泳涛，陈椿，张仁泉，陈克能，陈和忠，刘永煜，崔有斌，王允，庞烈文，于振涛，周鑫明，柳阳春，刘媛，方文涛，中国胸腺肿瘤协作组成员”。

2.第425页，第一作者梁光辉的中文作者单位应为“200032郑州，郑州大学附属肿瘤医院胸外科”。

特此更正。对此深表歉意。

Erratum: Comparison of the Masaoka-Koga and Te IASLC/ITMIG Proposal for Te TNM Staging Systems Based on the Chinese Alliance for Research in Tymomas (ChART) Retrospective Database

Guanghui LIANG, Zhitao GU, Yin Li, Jianhua FU, Yi Shen, Yucheng WEI, Lijie TAN, Peng ZHANG, Yongtao HAN, Chun CHEN, Renquan ZHANG, Ke-Neng CHEN, Hezhong CHEN, Yongyu LIU, Youbing CUI, Yun WANG, Liewen PANG, Zhentao YU, Xinming ZHOU, Yangchun LIU, Yuan LIU, Wentao FANG, Members of the Chinese Alliance for Research in Tymomas

Zhongguo Fei Ai Za Zhi, 2016, 19(7): 425-436.

In the version of this article initially published, errors appeared on page 425.

1. Te authors’ Chinese name list should be “梁光辉，谷志涛，李印，傅剑华，沈毅，魏煜程，谭黎杰，张鹏，韩泳涛，陈椿，张仁泉，陈克能，陈和忠，刘永煜，崔有斌，王允，庞烈文，于振涛，周鑫明，柳阳春，刘媛，方文涛，中国胸腺肿瘤协作组成员”.

2. Te frst author Guanghui LIANG’s Chinese author afliation should be “200032郑州，郑州大学附属肿瘤医院胸外科”.

## Notes

https://www.ncbi.nlm.nih.gov/pmc/articles/PMC6133977/

